# Deep Learning-Based Remaining Useful Life Estimation of Bearings with Time-Frequency Information

**DOI:** 10.3390/s22197402

**Published:** 2022-09-29

**Authors:** Bingguo Liu, Zhuo Gao, Binghui Lu, Hangcheng Dong, Zeru An

**Affiliations:** 1 School of Instrumentation Science and Engineering, Harbin Institute of Techonoloy, Harbin 150001, China; 2 Shanghai Spaceflight Precision Machinery Institute, Shanghai 201600, China

**Keywords:** bearing, short-time Fourier transform, remaining useful life, deep learning

## Abstract

In modern industrial production, the prediction ability of remaining useful life of bearings directly affects the safety and stability of the system. Traditional methods require rigorous physical modeling and perform poorly for complex systems. In this paper, an end-to-end remaining useful life prediction method is proposed, which uses short-time Fourier transform (STFT) as preprocessing. Considering the time correlation of signal sequences, a long and short-term memory network is designed in CNN, incorporating the convolutional block attention module, and understanding the decision-making process of the network from the interpretability level. Experiments were carried out on the 2012PHM dataset and compared with other methods, and the results proved the effectiveness of the method.

## 1. Introduction

In the operation of equipment, a large number of faults are caused by bearing failure. The prediction of remaining useful life (RUL) of bearings has become a key technology to ensure mechanical work safety. Existing bearing RUL prediction methods include two types, statistical life model prediction and data-driven prediction. Model-based methods include particle filtering [1], Eyring model [2], Weibull distribution [3], etc. These methods need a large number of statistical data as the basis to have certain reliability, but it is difficult to establish an accurate and general mathematical model for complex equipment.

The data-driven method uses the end-to-end training strategy and uses the state monitoring data to predict. A. Soualhi et al. proposed a rolling bearing condition monitoring method combining Hilbert–Huang transform, support vector machine, and support vector regression [4]. S.A. Aye et al. proposed an optimal Gaussian process regression (GPR) for low-speed bearing RUL [5]. Zhang Z. et al. converted these signals into the frequency domain using wavelet packet decomposition and fast Fourier transform and trained artificial neural network (ANN) [6].

In the field of CNN, G.S. Babu et al. and L. Ren et al., respectively, used fast Fourier transform and wavelet transform as pretreatments and used CNN to predict RUL [7,8]. Zhu et al. used the original vibration signal as the input of CNN for training and testing [9]. A.Z. Hinchi et al. used CNN and LSTM to predict RUL [10]. Biao Wang et al. made use of information by feedback connection that feeds back the output of the convolutional layer as input [11]. Xiang Li Wei et al. adopted the convolutional neural network of multi-scale feature fusion to gradually extract high-level features and discard low-level features at the same time. Features of different levels are fused, which improves the characteristic of one-way step-by-step transmission of feature information in the traditional convolutional network [12]. The difference between this paper and other methods is that the time-frequency diagram of vibration signals is taken as the network input, and CNN integrates LSTM and CBAM.

Focusing on RUL prediction, STFT pretreatment attention and memory-based CNN (SAL-CNN) is proposed in this paper. Based on CNN, the short-time Fourier transform (STFT) is used as the vibration signal preprocessing, and long short-term memory (LSTM) is integrated to make up for the fact that traditional CNN cannot consider the time correlation between vibration signal sequences. Moreover, convolutional block attention module (CBAM) is used to achieve the accurate acquisition of fault features and improve the accuracy of network life prediction.

The main contributions of this paper are as follows: Presenting a CNN framework (SAL-CNN), which takes STFT transformation as pretreatment and integrates CBAM and LSTM; visualizing the output of CBAM to explain the framework; discussing the relationship between RUL and frequency; and enhancing experimental credibility.

This paper introduces the basic theory about LSTM and CBAM in Section 2, the preprocessing algorithm and network SAL-CNN are detailed in Section 3, the experimental situation and comparative analysis are introduced in Section 4, and the interpretability of the network is subsequently studied. Finally, the summary is presented in Section 5.

## 2. Related Work

### 2.1. Long Short-Term Memory

**Figure 1:**
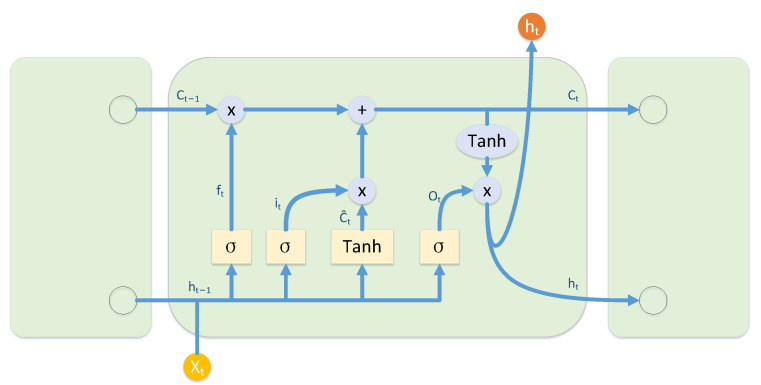
LSTM structures.

The recurrent neural network (RNN) [13] takes both the output characteristics of the last moment and data of the current moment as input. However, gradient disappearance and gradient explosion will occur with the increase of cycle layers. LSTM [14] is a special RNN, it introduces the concepts of gating unit and cell state. The input gate controls the output information from the previous layer, and the memory information from the previous moment is controlled by the forgetting gate. The most common LSTM structures are shown in Figure 1.

The expression of the forgetting gate is:

(1) $$f_{t}=\sigma \left(W_{f}\cdot \left[h_{t-1},x_{t}\right]+b_{f}\right)$$

The expression of the input gate is:

(2) $$i_{t}=\sigma \left(W_{i}\cdot \left[h_{t-1},x_{t}\right]+b_{i}\right)$$

(3) $$\tilde{C_{t}}=tanh\left(W_{C}\cdot \left[h_{t-1},x_{t}\right]+b_{C}\right)$$

The expression of the output gate is:

(4) $$o_{t}=\sigma \left(W_{o}\cdot \left[h_{t-1},x_{t}\right]+b_{o}\right)$$

(5) $$h_{t}=o_{t}\cdot tanh\left(C_{t}\right).,$$

where $W_{f},W_{i},Wc$, and $W_{o}$ represent the weight matrix, respectively, σ represents the sigmoid activation function, and acts on after passing through the forgetting gate and the input gate. The expression is:

(6) $$C_{t}=f_{t}\cdot C_{t-1}+i_{t}\cdot {\tilde{C}}_{t-1}.$$

### 2.2. Convolutional Block Attention Module

**Figure 2:**
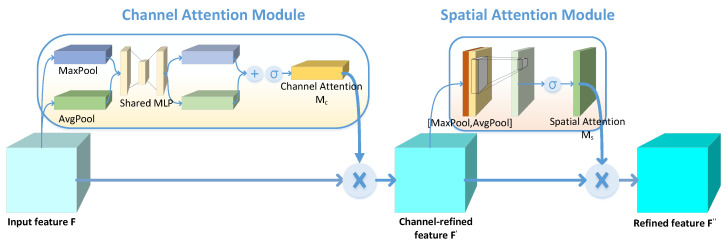
CBAM structures.

The convolutional block attention module (CBAM) [15] is a simple and effective injection module for feedforward convolutional neural network; its structure is shown in Figure 2. As a lightweight module, CBAM can be placed behind any feature graph as required. For feature map $F\in R_{c}\cdot h\cdot w$ of an intermediate layer, CBAM will deduce the 1-dimensional channel attention map $M_{c}\in R_{c}\cdot 1\cdot 1$ and 2-dimensional spatial attention map $M_{s}\in R1\cdot H\cdot W$, as shown below:

(7) $$F^{\prime }=M_{c}\left(F\right)⊗F$$

(8) $$F^{\prime \prime }=M_{s}\left(F^{\prime }\right)⊗F^{\prime },$$

where ⊗ is element-wise multiplication, and the expressions of the channel attention module *Mc*(*F*) and space attention module *Ms*(*F*) are as follows:

(9) $$M_{C}\left(F\right)=\sigma \left(\mathrm{MLP}\left(\mathrm{AvgPool}\left(F\right)\right)+\mathrm{MLP}\left(\mathrm{MaxPool}\left(F\right)\right)\right)$$

(10) $$M_{S}\left(F\right)=\sigma \left(f\left(\left[\mathrm{AvgPool}\left(F\right),\mathrm{MaxPool}\left(F\right)\right]\right)\right),$$

where σ is Sigmoid activation function, and *f* is convolution operation.

## 3. Framework

**Figure 3:**
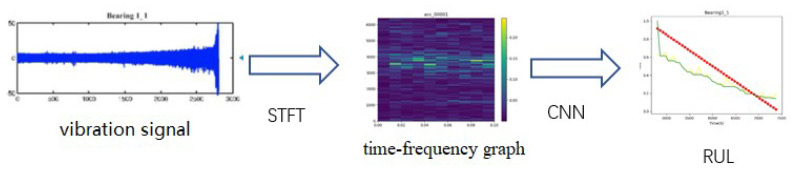
The flow diagram of our method.

Aiming at the problems that existing models cannot accurately predict RUL and work in real-time, an end-to-end detection framework, SAL-CNN, is proposed. The flow diagram is shown in Figure 3. The method includes two parts: vibration signal preprocessing and convolution neural network. CNN is designed to integrate the convolutional block attention module and long and short term memory network.

### 3.1. Vibration Signal Preprocessing

Since vibration signals have many spectral components and are non-stationary signals, the short-time Fourier transform [16] is used to preprocess the input data to obtain the time-frequency graph diagram. The short-time Fourier transform is a kind of joint time-frequency analysis method. Based on the Fourier transform, the whole segment of signal is processed by window segmentation, and each small segment is considered as a stationary signal. The window function slips along the time axis, and the Fourier transform is carried out in the neighborhood at any time.

For non-stationary signals $x\left(t\right)\in L^{2}\left(R\right)$, the short-time Fourier transform of $x\left(t\right)$ is expressed as:

(11) $$STFT_{x}\left(t,f\right)=\int_{-\infty }^{\infty }x\left(\tau \right)h\left(\tau -t\right)e^{-j2\pi ft}d\tau ,$$

where $h\left(\tau -t\right)$ is the window function.

Take the Fourier transform of $h\left(t\right)$: the energy is concentrated at the low frequency range, so it is usually thought of as a low-pass filter. As can be seen from Equation (11), the window function $h\left(t\right)$ moves along the time axis and conducts segmented processing on $h\left(t\right)$ in STFT transformation, the expression is:

(12) $$x_{t}\left(\tau \right)=x\left(\tau \right)h\left(\tau -t\right)$$

Later, take the Fourier transform of $x_{t}\left(\tau \right)$.

After windowing the signal, all the features of the signal covered by the window function will be displayed. The selection of the window function directly affects the results after STFT transformation. The frequency resolution is mainly determined by the main lobe width, so the window function with the narrowest main lobe and the smallest sidelobe peak value should be selected. The common window functions are rectangular window, triangular window, Hamming window, Blackman window, etc. and hamming window is selected as the window function comprehensively.

**Figure 4:**
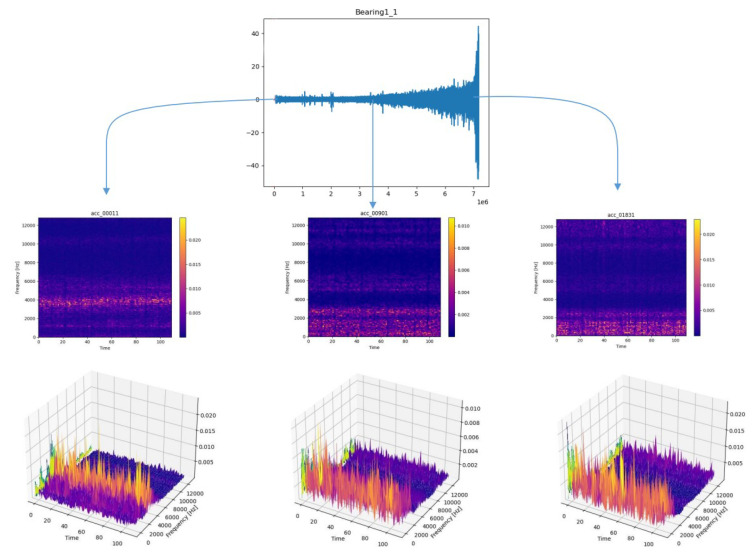
Three stages of vibration signal.

Figure 4 shows the vibration signal of bearing 1-1 in the whole life cycle and the time-frequency diagram after STFT transformation in the initial, middle, and final stages. The first row is the vibration signal diagram and the second row is the time-frequency diagram after STFT transformation. The *X*-axis represents the time axis, and the *Y*-axis represents the vibration frequency. The more brightly colored part represents the more concentrated energy of the vibration frequency in the same time series. The third row is a 3D display of the time-frequency graph, where the *z*-axis represents the frequency occurrence times. As can be seen from the figure, with the degradation of bearings and the increase of vibration signal amplitude, signals gradually concentrated in the low-frequency range. By this time, the high-frequency part was mainly an external noise signal, which made little contribution to life prediction. Therefore, the frequency of the upper part of the vibration signal was filtered out, and the remaining time-frequency information was used to predict RUL.

### 3.2. Network Model Structure

**Figure 5:**
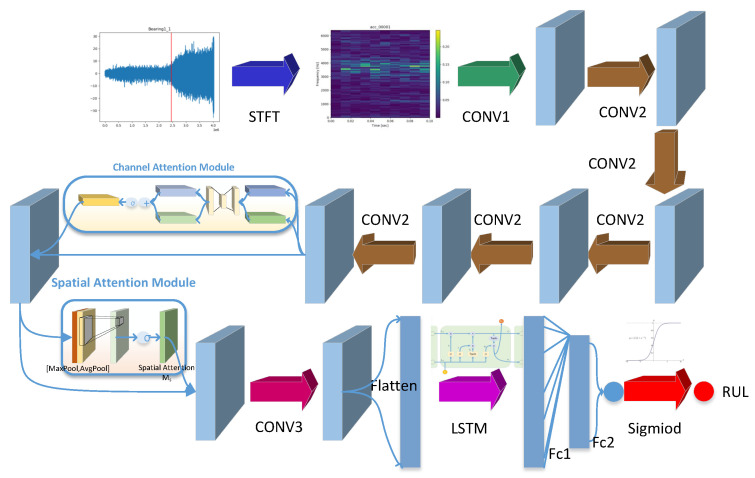
Structure of SAL-CNN.

Previous methods make use of only time-domain signals, which can increase the learning difficulty of deep learning models. Inspired by this, we propose to use the short-time Fourier transform to preprocess the input data, due to the fact that the short-time Fourier transform can provide information in both the time and frequency domains. It is worth mentioning that the fault information is often more obvious in the frequency domain. To be able to better extract temporal and spatial information, we add an attention mechanism module and an LSTM module to the ordinary convolutional neural network, which will enable the network to better extract these two types of information. Figure 5 shows the specific structure of our method.

Firstly, the vibration signal is preprocessed by STFT transformation (the size of the time-frequency diagram is 11 × 129 after preprocessing), enters into a six-layer convolution operation, and then into CBAM. After that, a one-layer convolution operation with a convolution kernel is used to compress the information, followed by LSTM. The last layer is a full connection layer and outputs a predicted life value; Table 1 describes specific network parameters.

## 4. Experiments and Discussions

### 4.1. Data Sets and Evaluation Indicators

In order to verify the effectiveness of the method, the data set of the 2012PHM Challenge [17] was used as the validation data set. The data set was provided by the FEMTO-ST research institute in France and was obtained by building an experimental platform in a laboratory environment [18]. Three working conditions were used for the experiments: (1) rotation speed:1800 r/min, radial force: 4000 N; (2) rotation speed: 1650 r/min, radial force: 4200 N; (3) rotation speed: 1500 r/min, radial force: 5000 N. The experimental platform collects vibration signals of the whole life cycle of bearings, and a total of 17 bearings are tested and their vibration signals collected. Table 2 shows the distribution of bearings.

In order to quantitatively evaluate the network structure, *MAE* is used as the evaluation index. As shown by expression 13, the more accurate the prediction results are, the lower *MAE* is:

(13) $$MAE=\frac{1}{s}\sum_{i=1}^{s}\left({\mathrm{ActRUL}}_{i}-{\mathrm{PreRUL}}_{i}\right),$$

where *S* is the number of testing samples, ${\mathrm{ActRUL}}_{i}$ is the actual RUL values corresponding to the *i*-th testing sample, and ${\mathrm{PreRUL}}_{i}$ is the predictive RUL values corresponding to it.

### 4.2. Experimental Setup

All bearings were used as prediction bearings in turn, and the remaining 16 bearings were used as training sets in each experiment to obtain cross-validation results.

The training set is input into SAL-CNN for training, SAL-CNN is implemented by PyTorch with Nvidia GeForce GTX 2080ti GPU, the iteration training is terminated after 150 epochs. The loss function is L1 loss and the optimizer is Adam, the learning rate is set to 0.001 and batch size is 32. The dropout rate is 0.1. The number of LSTM cycle layers is 1.

### 4.3. Analysis

**Figure 6:**
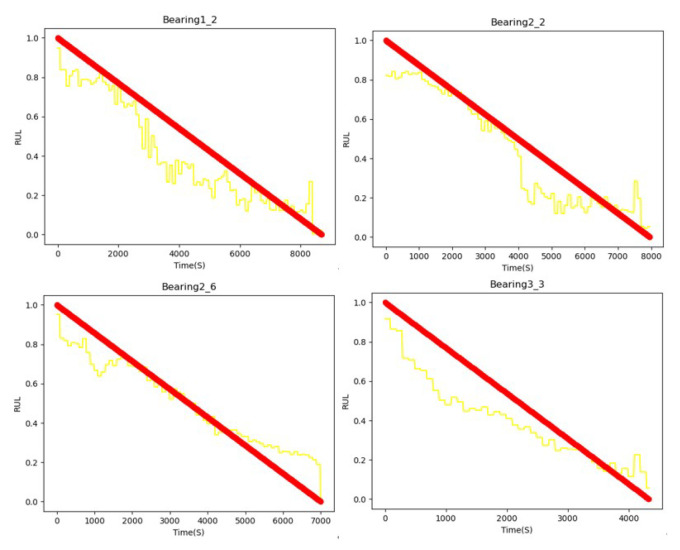
The RUL prediction results of selected bearings.

Figure 6 shows the prediction results of selected bearings, which is under different work conditions. The red curve is the real RUL and the yellow curve is the predicted RUL. It can be seen that the yellow curve basically fits the true value.

This paper conducted comparative experiments with four methods on the 2012PHM Challenge data set. Table 3 shows MAE indexes compared with other methods. DNN is also known as multi-layer perceptron (MLP), single scale-low (SSL), and single scale-high (SSH) using low-scale features and high-level features, respectively, for RUL estimation without feature concatenation, and a multi-scale method integrating an all scale feature [12].

The comparison result indicates that the MAE obtained by the proposed algorithm is higher than others. Among the 17 bearings, the prediction results of 11 bearings are the best, and 2/3 of the results are better and evenly distributed under each working condition, indicating that this method is effective for bearings under three working conditions. According to the average value of the final MAE, it can be seen that the results in this paper are improved by 2.8 compared with the multi-scale method.

### 4.4. Feature Information Visualization

Feature visualization is a post-hoc interpretation of the pre-training model. After training, the decision-making process of CNN is explained in the form of images, which can intuitively reflect the key areas of input features that the network focuses on.

Table 4 shows the time-frequency graph and heatmap of bearings 1-1, 1-3, 1-5 in the early, middle, and late stages. In time-frequency graph, the ordinate is the frequency and the value range is [0, 6400], and the abscissa is time and the value range is [0, 1]. In the trained network it is possible to visualize the output of the CBAM layer and obtain aheatmap where the highlighted parts represent the areas that the network is more concerned with. As time goes by, the frequency concentrated range in the time-frequency graph is from 3000–4000 Hz to 0–2000 Hz, and the high-frequency part is basically no energy. It can also be seen from the heatmap that the network pays more attention to the information of the low-frequency part in the late stage, it means low-frequency signal may become more import.

## 5. Summary

This paper proposes a long and short-term memory convolutional neural network that introduces a convolutional block attention module. The improved algorithm makes up for the lack of traditional convolutional networks that cannot consider the time correlation of vibration signal sequences. At the same time, the convolutional block attention module further improves the prediction performance of the network. The test on the PHM2012 data set proves that the average MAE is slightly higher than the best recent results.

In addition, we found that for predicting the RUL task, low-frequency vibration signals may be more worthy of attention at a later stage; the time-frequency graph directly shows that most of the energy is distributed in the low-frequency range at a later stage. Using heatmap to visualize the output of CBAM, we also found that the network at the later stage pays more attention to the low-frequency range, and interpretability confirms this conjecture.

In the future, we will explore the use of multimodal information to leverage both time and frequency domain information to further improve the prediction of bearing life. In addition, we believe that the study of deep learning interpretability to obtain human-understandable knowledge may provide more insight into the field.

## Figures and Tables

**Table 1:** Architecture of SAL-CNN.

Layer	Parameter	Value
STFT	Window	Hamming
	fs	25,600 Hz
	Nperseg	512
Conv1	In channels	1
	Kernel size	3 × 3
	Out channels	5
Conv2	In channels	5
	Kernel size	3 × 3
	Out channels	5
Conv3	In channels	5
	Kernel size	3 × 3
	Out channels	1
LSTM	Input size	1408
	Hidden size	1408
	Num layers	2

**Table 2:** Distribution of bearings in 2012PHM Challenge.

Operating Mode	Radial Force (N)	Rotate Speed (rpm)	Bearing
1	4000	1800	Bearing1-1, Bearing1-2
			Bearing1-3, Bearing1-4
			Bearing1-5, Bearing1-6, Bearing1-7
2	4200	1650	Bearing2-1, Bearing2-2
			Bearing2-3,Bearing2-4
			Bearing2-5, Bearing2-6, Bearing2-7
3	5000	1500	Bearing3-1, Bearing3-2, Bearing3-3

**Table 3:** Comparison of life prediction results based on MAE index. Lower MAE means better performance.

Bearing	DNN	SSL	SSH	Multi-Scale [12]	SAL-CNN
Bearing1-1	30.4	24.3	24.4	21.4	**16.9**
Bearing1-2	28.5	17.4	22.4	14.7	**13.0**
Bearing1-3	16.3	9.5	9.3	**7.8**	10.3
Bearing1-4	32.1	**21.3**	24.5	21.8	23.0
Bearing1-5	28.7	21.4	19.5	18.5	**11.8**
Bearing1-6	32.4	24.5	20.4	**20.3**	21.4
Bearing1-7	16.6	9.6	11.7	**8.3**	11.9
Bearing2-1	28.6	24.2	25.2	21.5	**18.5**
Bearing2-2	26.4	15.3	16.5	16.2	**8.8**
Bearing2-3	40.5	35.2	31.5	32.8	**14.8**
Bearing2-4	22.3	11.6	9.9	**6.1**	24.2
Bearing2-5	31.4	27.3	25.2	23.6	**23.3**
Bearing2-6	29,4	23.2	19.8	20.7	**8.6**
Bearing2-7	38.0	28.2	28.4	27.4	**27.1**
Bearing3-1	28.3	28.5	26.3	**19.6**	25.2
Bcaring3-2	30.4	25.2	23.7	23.1	**18.1**
Bearing3-3	38.5	35.4	33.5	30.4	**11.1**

**Table 4:** Spectrogram and heatmap of bearings in three stages.

Bearing	Image Type	Early Stage	Middle Stage	Late Stage
1-1	Time-frequency graph	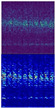	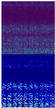	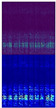
	Heatmap			
1-3	Time-frequency graph	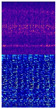	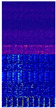	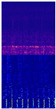
	Heatmap			
1-5	Time-frequency graph	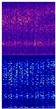	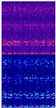	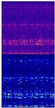
	Heatmap			

## Data Availability

Not applicable.

## References

[1] Jouin M., Gouriveau R., Hissel D., Péra M.C., Zerhouni N. (2016). Particle filter-based prognostics: Review, discussion and perspectives. Mech. Syst. Signal Process..

[2] Jouin M., Gouriveau R., Hissel D., Pera M.C., Zerhouni N. (2016). Degradations analysis and aging modeling for health assessment and prognostics of PEMFC. Reliab. Eng. Syst. Saf..

[3] Ali J.B., Chebel-Morello B., Saidi L., Malinowski S., Fnaiech F. (2015). Accurate bearing remaining useful life prediction based on Weibull distribution and artificial neural network. Mech. Syst. Signal Process..

[4] Soualhi A., Medjaher K., Zerhouni N. (2014). Bearing Health monitoring based on Hilbert-Huang Transform, Support Vector Machine and Regression. IEEE Trans. Instrum. Meas..

[5] Aye S.A., Heyns P.S. (2017). An integrated Gaussian process regression for prediction of remaining useful life of slow speed bearings based on acoustic emission. Mech. Syst. Signal Process..

[6] Zhang Z., Wang Y., Wang K. (2013). Fault diagnosis and prognosis using wavelet packet decomposition, Fourier transform and artificial neural network. J. Intell. Manuf..

[7] Babu G.S., Zhao P., Li X.L. Deep Convolutional Neural Network Based Regression Approach for Estimation of Remaining Useful Life. Proceedings of the International Conference on Database Systems for Advanced Applications.

[8] Ren L., Sun Y., Wang H., Zhang L. (2018). Prediction of Bearing Remaining Useful Life with Deep Convolution Neural Network. IEEE Access.

[9] Zhu J., Nan C., Peng W. (2018). Estimation of Bearing Remaining Useful Life based on Multiscale Convolutional Neural Network. IEEE Trans. Ind. Electron..

[10] Hinchi A.Z., Tkiouat M. (2018). Rolling element bearing remaining useful life estimation based on a convolutional long-short-term memory network. Procedia Comput. Sci..

[11] Wang B., Lei Y., Yan T., Li N., Guo L. (2020). Recurrent convolutional neural network: A new framework for remaining useful life prediction of machinery. Neurocomputing.

[12] Li X., Zhang W., Ding Q. (2019). Deep learning-based remaining useful life estimation of bearings using multi-scale feature extraction. Reliab. Eng. Syst. Saf..

[13] Elman J.L. (1990). Finding Structure in Time. Cogn. Sci..

[14] Zaremba W., Sutskever I., Vinyals O. (2014). Recurrent Neural Network Regularization. arXiv.

[15] Woo S., Park J., Lee J.Y., Kweon I.S. CBAM: Convolutional Block Attention Module. Proceedings of the European Conference on Computer Vision.

[16] Chui C.K., Jiang Q., Li L., Lu J. (2021). Analysis of an adaptive short-time Fourier transform-based multicomponent signal separation method derived from linear chirp local approximation. J. Comput. Appl. Math..

[17] Sutrisno E., Oh H., Vasan A.S.S., Pecht M. Estimation of remaining useful life of ball bearings using data driven methodologies. Proceedings of the 2012 IEEE Conference on Prognostics and Health Management.

[18] Nectoux P., Gouriveau R., Medjaher K., Ramasso E., Chebel-Morello B., Zerhouni N., Varnier C. PRONOSTIA: An experimental platform for bearings accelerated degradation tests. Proceedings of the IEEE International Conference on Prognostics and Health Management, PHM’12.

